# Case report: Toripalimab plus anlotinib in postoperative recurrent renal pelvic sarcomatoid urothelial carcinoma

**DOI:** 10.3389/fonc.2024.1397855

**Published:** 2024-09-25

**Authors:** Xinrong Hu, Lin Deng, Yufei Pan, Guozhen Zhang, Xiaolan Ruan, Xinlan Jiang, Hao Shen, Lei Zhao, Jun Dong

**Affiliations:** ^1^ Department of Oncology, Nanxishan Hospital of Guangxi Zhuang Autonomous Region, Guilin, China; ^2^ Department of Pathology, Nanxishan Hospital of Guangxi Zhuang Autonomous Region, Guilin, China; ^3^ Department of General Medicine, Sun Yat-sen University Cancer Center, Guangzhou, China

**Keywords:** toripalimab, anlotinib, postoperative recurrence, renal pelvic urothelial carcinoma, upper-tract urothelial carcinoma, sarcomatoid urothelial carcinoma, immune checkpoint inhibitor, tyrosine kinase inhibitor

## Abstract

**Background:**

Sarcomatoid urothelial carcinoma (SUC) is a rare renal malignancy. Its biological malignancy is high, the prognosis is poor, diagnostic and treatment options are few, and there is no standard treatment plan.

**Case presentation:**

In this case, a 64-year-old woman was hospitalized with fever and lower back pain one week previously. The preliminary diagnosis was a right kidney stone with a urinary tract infection. After the anti-infection treatment, a percutaneous right nephrostomy was performed. The intraoperative biopsy (renal pelvis) finding was infiltrating urothelial carcinoma with a sarcomatoid variation. Subsequently, radical surgery was performed for cancer of the right renal pelvis. Implant metastasis of the abdominal wall and adjacent abdominal cavity occurred half a month after the surgery. The lesion was resected again, and two cycles of doxorubicin plus carboplatin chemotherapy were administered. However, the disease progressed more rapidly after the chemotherapy. With the written consent of the patient, the treatment was altered to targeted immune therapy with toripalimab plus anlotinib. A clinical cure was achieved after nine cycles of treatment with no obvious lesions on imaging. The maintenance therapy was administered consecutively for over a year, and the patient is at present still in good condition with a disease-free survival exceeding two years.

**Conclusion:**

This case proves that the combination of toripalimab and anlotinib is effective in the treatment of recurrent renal SUC. To the best of our knowledge, this is the first reported case of a patient with advanced recurrent urothelial carcinoma of the renal pelvis sarcomatoid cured with this therapy.

## 1 Introduction

Primary tumors in the renal pelvis are rare and account for 7% of all renal tumors. Most of them are the high-grade urothelial carcinoma (UC) (1). Urinary tract epithelial carcinoma can exhibit multiple tissue or cell mutations and metaplasia, such as squamous cell carcinoma, adenocarcinoma, papillary carcinoma, sarcomatoid carcinoma, and lymphoepithelioma-like subtype. Of these, sarcomatoid urothelial carcinoma (SUC) belongs to one of the subtypes of rare invasive UC (2). SUC mostly occurs in the bladder and accounts for approximately 0.1%–0.3% of all the UC (3), and SUC occurring in the renal pelvis is even rarer. At present, as few as just over 20 cases have been reported at home and abroad. SUC originates from the epithelial tissue and displays the characteristics of sarcoma. Its etiology remains unclear. It may originate from monoclonal tumor cells with epithelial and mesenchymal components, and its occurrence may be related to gene amplification of chromosomes 3, 7, and 17 and deletion of chromosome 9p21 (4). In this case report, a 64-year-old woman with postoperative SUC was treated with toripalimab plus anlotinib for nine cycles and achieved an excellent outcome without recurrence for about two years.

## 2 Case presentation

**Figure 1:**
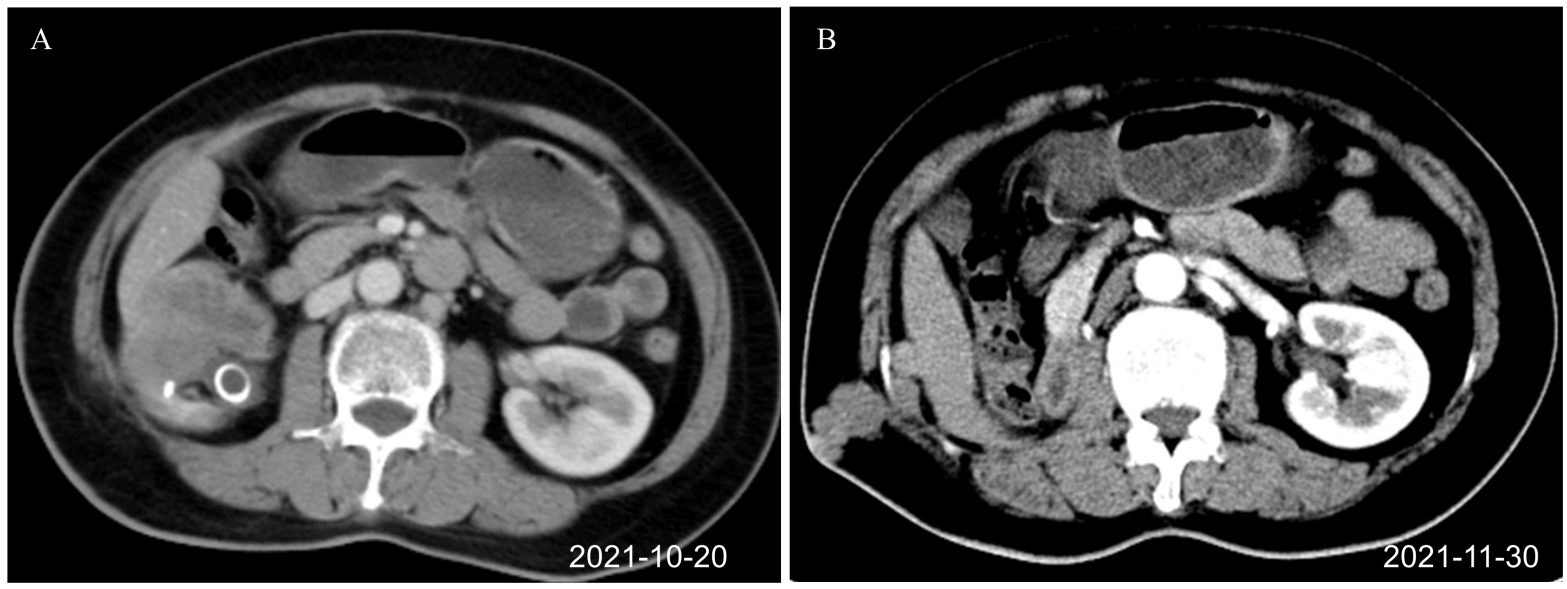
Renal CT images before combination therapy of toripalimab plus anlotinib (axial plane). **(A)** Right renal pelvis tumor before surgery, J-tube drainage placed in the external hospital (October 20, 2021). **(B)** Recurrence after the first operation. Tumors were seen at the scar of the drainage tube operation and around the liver (November 30, 2021). CT, computed tomography.

**Figure 2:**
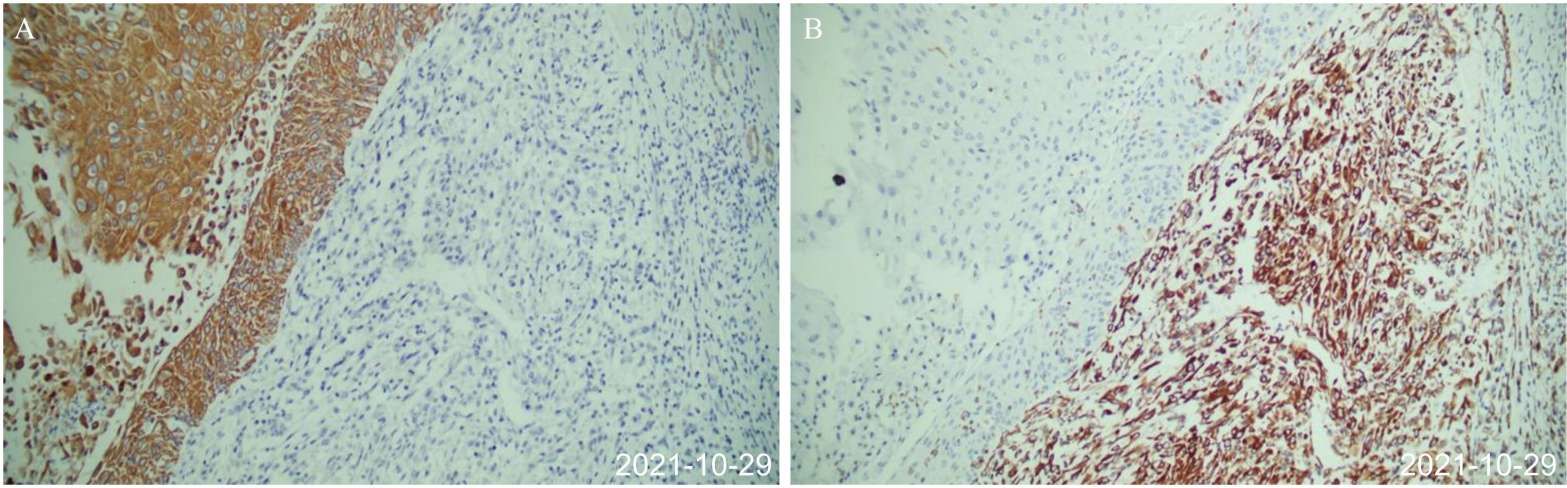
Postoperative pathology after first surgery (October 29, 2021). **(A)** The epithelial-derived component was positive for immunohistochemical CK. **(B)** Immunohistochemical vimentin of mesenchymal tissue-derived components were positive, and the cells were spindle-shaped.

The patient is a 64-year-old woman. In September 2021, she was hospitalized with fever and lower back pain for one week. Laboratory test results were as follows: white blood cell count 10.53 × 10^9^ cells/L, hemoglobin 103 g/L, urinary leukocyte (2+), urinary occult blood (2+), urinary protein (1+), normal liver, renal, and prothrombin time function, alpha-fetoprotein 1.07 ng/ml. She was initially diagnosed with stones in the right kidney. Percutaneous puncture and right nephrostomy were performed on September 18, 2021. Percutaneous nephroscopic lithotripsy of right kidney stones and percutaneous pyeloscopic biopsy of renal pelvis masses were performed on September 26, 2021. The postoperative pathology revealed that a few mucosal cells exhibited chronic suppurative inflammation with squamous metaplasia and moderate atypical hyperplasia. Hence, malignancy could not be ruled out. However, the patient refused to undergo radical surgery for renal pelvis cancer. On October 8, 2021, percutaneous nephrolithotripsy and biopsy of the right renal pelvis tumor was repeated. Postoperative pathology and immunohistochemistry (IHC) suggested invasive UC (sarcomatoid variant). Contrast-enhanced computed tomography (CT) of the abdomen demonstrated a right kidney occupying space, and the possibility of neoplastic lesions (renal pelvis cancer) was considered with multiple stones in the right kidney (**Figure 1A**). After the right double J tube operation, robot-assisted laparoscopic right radical surgery for renal pelvis cancer was performed under general anesthesia on October 22, 2021. Postoperative pathology (October 29, 2021) of the right kidney was as follows: (1). A malignant tumor was present, which comprised two components. Infiltrating high-grade UC with squamous differentiation was predominant, and a small amount of spindle cell sarcoma was observed. The tumor size was approximately 5 cm × 4 cm × 4 cm, and it was located in the renal pelvis. No definite nerve invasion or vascular tumor thrombus was found. IHC indicated the following: The cancer characteristics were CK(+), Vim(−), CK20(−), GATA-3(−), P63(+) 70%–80%, and Ki67(+) 30%–40%. The sarcoma composition was CK(−), Vim(+), SMA(−), S-100(−), CD34(−), P63(−), and Ki67(+) approximately 50%. HER-2 (0), MLH1 (+), MSH2 (+), MSH6 (+), PMS2 (+); the results indicate proficient mismatch repair (pMMR), suggesting a microsatellite stable (MSS) phenotype, with a very low likelihood of high microsatellite instability (MSI-H). (2). The perirenal fat sac was not invaded by the tumor. (3). Tumor was not found at the broken end of the ureter. (4). Some areas of the surrounding renal tissue exhibited renal pelvis expansion, renal parenchymal thinning, nephron reduction, focal calcification, and chronic infiltration of inflammatory cells (**Figure 2**).

Half a month after the operation, there was a lump in the scar of the original right lumbar percutaneous puncture drainage tube, which the patient was able to touch by herself. The size of the lump increased progressively. CT examination of the lung and abdomen on November 30, 2021, showed the absence of metastasis in the lung. Multiple nodules were present on the right abdominal wall and adjacent abdominal cavity. Postoperative implantation and metastasis of the tumor were considered (**Figure 1B**). After multidisciplinary discussion, the decision was made to perform surgical resection of the lesion before adjuvant chemotherapy. Resection of the tumor in the right abdominal wall and the abdominal cavity was performed on December 3, 2021. Postoperative pathology (December 12, 2021) revealed lumbar and retroperitoneal masses, and spindle cell sarcoma was considered. Microscopically, a small number of inflammatory cells infiltrated the tumor stroma. The tumor involved the dermis. The lateral and basal incisional margins of the skin were clear. The tumor was close to the cutting edge of the base. Combined with the clinical history, the patient was diagnosed to have metastatic sarcomatoid carcinoma of the renal pelvis. IHC revealed CK (−), Vim (+), CK20(−), GATA3(−), P40(−), CA9(−), CD34(−), P53(−), and Ki67(+) approximately 70%. After the surgery, the patient was given two cycles of chemotherapy (doxorubicin plus carboplatin) on December 15, 2021, and February 5, 2022, for renal pelvis SUC. Evaluation of the curative effect after two cycles of chemotherapy disease progression showed that the disease had progressed rapidly. There is no standard treatment plan for postoperative recurrent renal pelvis SUC. The Chinese Society of Clinical Oncology (CSCO) guidelines of 2021 recommend immunotherapy as the second-line treatment for recurrent UC. Second-line targeted therapy is recommended for unresectable soft tissue sarcoma. Based on clinical experience, targeted immunotherapy involving toripalimab 240 mg on Day 1 combined with anlotinib 12 mg on Days 1–14 was given. The treatment was repeated for three weeks.

**Figure 3:**
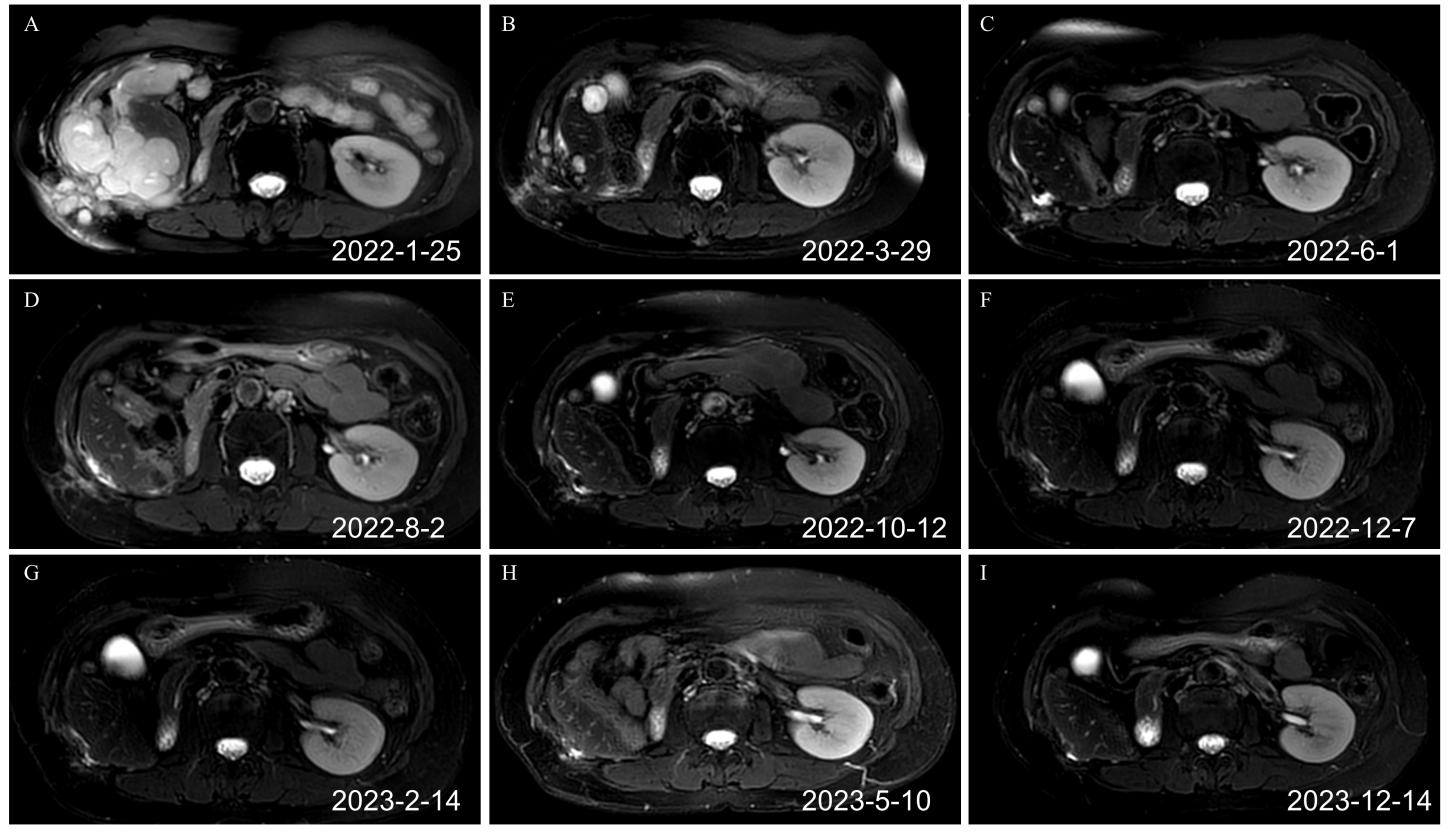
Renal MRI T2 images before and after the combination of toripalimab and anlotinib (axial plane). **(A)** Recurrence after the second operation before the immunotherapy. Tumors were detected in the liver, abdominal cavity, and abdominal wall, which had fused into one piece (February 25, 2022). **(B)** After three cycles of targeted immunotherapy, most tumors of the liver, the abdominal cavity, and the abdominal wall disappeared (March 29, 2022). **(C)** After six cycles of targeted immunotherapy, the tumors of the liver, the abdominal cavity, and the abdominal wall basically disappeared (June 1, 2022). **(D)** After nine cycles of targeted immunotherapy, the tumors of the liver, the abdominal cavity, and the abdominal wall basically disappeared (August 2, 2022). **(E)** After 12 cycles of targeted immunotherapy (October 12, 2022). **(F)** After 14 cycles of targeted immunotherapy (December 7, 2022). **(G)** After 16 cycles of targeted immunotherapy (February 14, 2023). **(H)** After 19 cycles of targeted immunotherapy (May 10, 2023). **(I)** Renal MRI image on December 14, 2023. MRI, magnetic resonance imaging.

**Figure 4:**
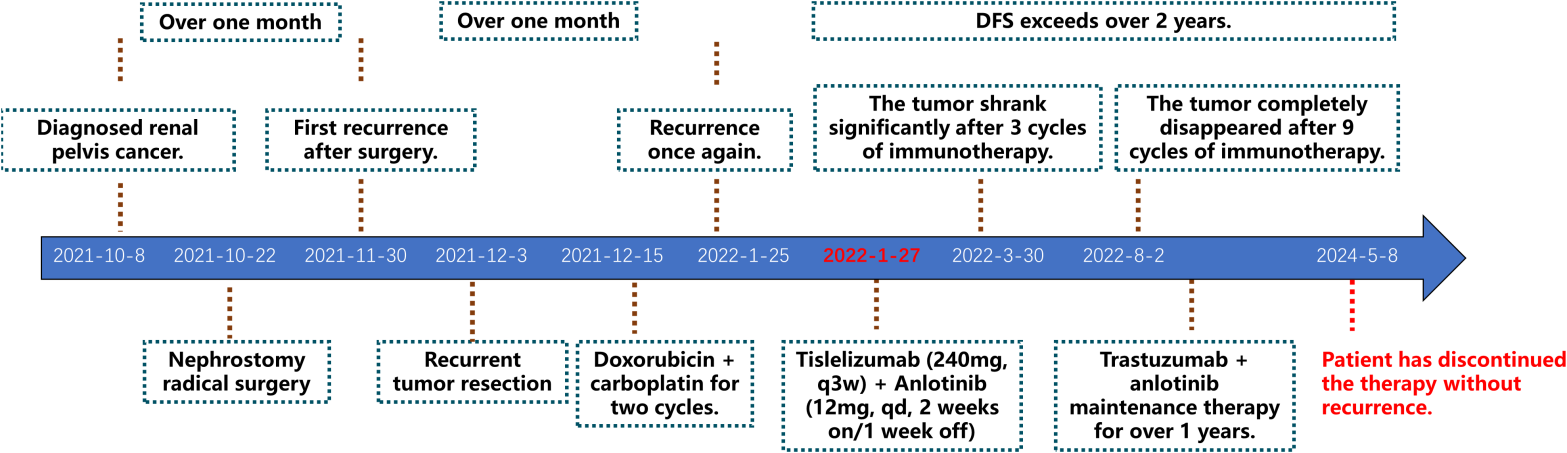
Treatment timeline. DFS, disease-free survival.

The first targeted immunotherapy commenced on January 27, 2022. After two cycles of treatment, the patient felt that the abdominal discomfort had disappeared. After three cycles of treatment, a review on March 29, 2022, using abdominal magnetic resonance imaging (MRI) indicated that most of the lesions in the abdominal wall, perihepatic region, and abdominal and pelvic cavity observed during the original operation had disappeared. After six cycles of treatment, the abdominal and pelvic tumors had disappeared. After nine cycles of targeted immunotherapy, there was no obvious tumor focus on imaging. The patient is in good condition and has achieved a clinical cure. At present, two years after treatment, no tumor recurrence was found in the follow-up examination (**Figure 3**; **Supplementary Figures S1**, **S2**). The disease-free survival (DFS) exceeds two years. The patient has discontinued medication treatment and further follow-up was performed as required. On May 8, 2024, a follow-up MRI indicated no signs of disease recurrence (**Supplementary Figure S3**). The treatment timeline is illustrated in **Figure 4**.

## 3 Discussion

### 3.1 Clinical characteristics

The age of onset of renal pelvis SUC is >50 years. The ratio of males to females is 2–3:1 (5). The exact cause of the disease remains unclear. According to a relevant research report, it may be closely related to smoking, radiation, and cyclophosphamide exposure (6). This patient had a history of kidney stones over several decades. She had a history of fever and lower back pain and had undergone anti-infection treatment several times. The adverse stimulation of repeated stones combined with infection was considered, as it might have been a pathogenic factor for renal pelvis SUC. The clinical manifestations of renal pelvis SUC are not highly specific and are similar to those of UC of the renal pelvis. The main clinical manifestations are painless gross hematuria, lower back pain, abdominal mass, and hydronephrosis. Of these, hematuria is the most common manifestation (7). In this case, some symptoms were masked owing to the presence of stones and infection. The imaging manifestations of SUC are also not typical and are prone to being misdiagnosed. Color ultrasonography of the urinary system, intravenous pyelography, CT, lower urinary tract imaging, MRI, and other imaging examinations have great significance in displaying the size, location, and shape of the tumors. These methods can aid in identifying whether the tumor has invaded the surrounding tissues and in detecting local lymph node enlargement. However, these methods have little guiding significance in identifying the tumor types (8).

### 3.2 Diagnosis and differential diagnosis

Histopathological examination and immunohistochemical analysis are the gold standards for the diagnosis of SUC. Endoscopic puncture biopsy contains fewer tumor tissue components because most sarcomatoid components are located in the deep part of the tumor. Therefore, SUC is often misdiagnosed as simple UC (9). Radical surgery can yield adequate specimens to help determine the diagnosis. The pathological features of renal pelvis SUC are extensive infiltration of tumor cells into the renal parenchyma, hemorrhage, necrosis, and sarcomatous reaction in the stroma. These features make the kidney appear to be diffusely swollen. Histologically, SUC presents various cell morphologies. Cancerous and sarcomatoid tissues can appear simultaneously. The key manifestations of cancerous tissues are squamous cell carcinoma, large cell carcinoma, and small cell carcinoma. The sarcomatoid tissue is predominantly a pleomorphic cell tumor, mainly spindle cell; hence, sarcomatoid carcinoma is also called spindle cell carcinoma (10). With regard to the immunophenotypes, CK is diffusely expressed in cancer tissues and partially or focally expressed in sarcomas. The expression of CK in sarcomas is low, and vimentin is diffusely expressed.

In addition to the common UC of the urinary system, the differential diagnosis of SUC should also be considered and the condition should be differentiated from carcinosarcoma. The concept of carcinosarcoma was first proposed by Dent (11) in 1955. Carcinosarcoma comprises epithelial and mesenchymal components, namely cancer and sarcoma. Urinary system SUC and carcinosarcoma are categorized into three tissue types: 1) The tumor is derived from the epithelial tissue. There are two types of morphologies, namely, epithelial and mesenchymal tissue. 2) The tumor contains malignant elements derived from the epithelial and mesenchymal tissues. 3) The tumor is chiefly composed of a malignant element of epithelial origin but is also accompanied by mesenchymal elements that make it malignant (12). The latter two are called ‘true carcinosarcomas’, while the first is commonly called sarcomatoid carcinoma. That is, sarcomatoid carcinoma tissue cells are derived from epithelial tissue, but morphologically, there are two forms of differentiation to epithelial tissue and mesenchymal tissue. Histologically, carcinosarcoma contains malignant elements derived from both epithelial and mesenchymal tissues (12). In sarcomatoid carcinoma, spindle sarcomatoid cells express both CK and vimentin, whereas the sarcomatous components in carcinosarcoma do not express CK (13).

### 3.3 Treatment

The best treatment for renal pelvis SUC is yet to be determined. Currently, early diagnosis and early radical surgery remain the most important means to prolong the survival time and improve the prognosis of patients (6, 7). We believe that radical nephroureterectomy is the best surgical modality, and efforts should be made to keep the tumor intact during the operation. If the tumor is damaged, intraperitoneal implantation metastasis can easily occur. Moreover, preoperative puncture examination and J tube drainage may cause tumor peritoneal implantation metastasis. In our patient, the two recurrence sites were at the scar of the drainage tube and sustained an external hospital puncture. Therefore, the recurrence after the two surgical procedures could be related to the repeated percutaneous punctures before the operation.

No standard treatment is available for recurrent renal pelvis SUC. Most reports published to date suggest that sarcomatoid carcinoma is not sensitive to chemotherapy or radiotherapy. The clinical value of other adjuvant therapies, including systemic chemotherapy and radiotherapy, is also limited, and the curative effect is uncertain (14). According to some studies, adjuvant chemotherapy can reduce the recurrence rate to a certain extent; however, its impact on the survival rate of patients is unclear (15). At Johns Hopkins Medical Institution in the United States, radical surgery is preferred for treating patients with SUC. Post-surgically, cisplatin, gemcitabine, and docetaxel are administered as adjuvant chemotherapy. For patients with the pathology of squamous cell carcinoma, the use of a combination of drugs (cisplatin plus etoposide) as adjuvant chemotherapy after the surgery is recommended (16). However, in China, there is a lack of consensus on this adjuvant treatment scheme.

Our patient was treated with radical surgery, and recurrence was seen less than one month after the surgery. If the tumor relapses, the recurrent focus is removed surgically, and adjuvant chemotherapy is administered after the operation. However, active local treatment (surgery) combined with systemic treatment (chemotherapy) after recurrence is not likely to benefit the patient. A re-examination of the imaging showed that the lesions were significantly more diffuse than the ones before the treatment. Moreover, the number of lesions had increased, and the disease progressed rapidly. This finding confirmed the previously reported characteristics of renal pelvis SUC, such as high malignancy, strong invasion, poor chemotherapeutic effect, and poor prognosis.

Currently, molecular targeted therapy and immunotherapy have gradually assumed significance in the treatment of sarcomatoid renal cell carcinoma (especially in the postoperative adjuvant therapy for metastatic renal cell carcinoma). Nonetheless, its clinical application in renal pelvis SUC has not been reported. Immune checkpoint inhibitors (ICIs) can prevent the immune escape of the tumor and then produce resistance to the tumor (17, 18). Studies highlighted the association of SUC with the overexpression of PD-L1 (19), which also been demonstrated a significant correlation with tumor immune infiltration in SUC (20). The overexpression of PD-L1 is typically associated with the infiltration of CD8(+) T cells in tumors (19, 20). Anti-PD-L1 therapy may shift the functionality of tumor-associated macrophages from an immunosuppressive to an immune-activating state, resulting in heightened cytotoxicity of activated CD8(+) T cells (21). In recent years, several clinical trials have confirmed the pertinent role of ICIs in the treatment of locally advanced or metastatic UC (22–25). Therefore, ICIs have been approved for use in the second-line treatment of advanced UC (26).

In addition to immune infiltration, epithelial-to-mesenchymal transition (EMT) is also associated with PD-1/PD-L1. A positive correlation exists across all cancer types between the expression of immune checkpoint genes including PD-1 and EMT (27), which (type III EMT) occurs during the metastatic process of cancer cells with some tumor cells simultaneously expressing both epithelial and mesenchymal markers (16). This hybrid state of partial EMT is associated with increased cellular plasticity, endowing them with the ability to adapt, survive, and disseminate effectively (28), along with the impact on the efficacy of therapeutics (28, 29). EMT intricately involves dynamic alterations in the intracellular cytoskeleton and extracellular matrix. An elongated morphology of tumor cells following EMT was facilitated by the formation of actin-driven protrusions and upregulation of vimentin expression (30). A study focusing on UC of the bladder unveiled that increased expression of mesenchymal markers and actin-cytoskeleton regulators correlated with pathological progression during cisplatin-based neoadjuvant chemotherapy (NAC). Overexpression of N-cadherin was predictive for disease-specific mortality (31). The findings suggested the role of EMT in cisplatin resistance during NAC in UC. Some EMT even processes exhibit inducible stem cell-like properties, thereby providing robust support for cancer cell survival (32). The SUC cells overexpressed PD-L1, with upregulated vimentin and downregulated E-cadherin (16). Therefore, the application of ICIs in SUC may simultaneously suppress EMT, potentially enhancing the overall antitumor effect.

The introduction of ICIs has significantly expanded the therapeutic horizons for patients with metastatic UC. Nevertheless, only a subset of patients experience sustained responses, with response rates remaining below 20% in the context of first-line treatment or beyond chemotherapy (33). Preclinical studies have indicated that tyrosine kinase inhibitors (TKIs) could potentially enhance the efficacy of immunotherapeutic agents within the tumor microenvironment (34). The rationale for using TKIs in UC lies in their ability to target oncogenic drivers, such as fibroblast growth factor receptor (FGFR) mutations or fusions, which are prevalent in certain molecular subtypes of UC and are associated with resistance to ICIs (33). For sarcomatoid differentiated UC that recurred after surgery as first-line therapy, there has been case report of still favorable outcomes (35). TKIs have emerged as a pivotal therapeutic strategy in the treatment of UC, particularly in the context of advanced or metastatic disease (36). Wang et al. (17) observed the epidermal growth factor receptor is positive in SUC cells. Therefore, it is speculated that TKIs may be effective in treating recurrent SUC.

Toripalimab is a full-human monoclonal antibody against the PD-1 receptor. The drug can prevent PD-1 of T lymphocytes from binding to PD-L1 on the surface of tumor cells by blocking them. In addition, toripalimab can relieve the immunosuppressive effect of tumor cells on immune cells and make immune cells play the role of antitumor cells and kill tumor cells. Anlotinib is a small molecule multitarget TK inhibitor. The targets include VEGFR1/2/3, FGFR1/2/3, PDGFR α/β, C-Kit, and RET. After two cycles of medication, the patient felt that the abdominal discomfort was significantly relieved. Most tumors had disappeared after three cycles, and all tumors had disappeared from the patient’s imaging examination after six cycles. After nine cycles, there was no obvious tumor focus on imaging, and the tumor was clinically cured. The curative effect was quite obvious. Based on these findings, it could be theorized that SUC may contain the target of the above-targeted drugs. Moreover, PD-L1 or tumor mutation burden (TMB) may be highly expressed. During the treatment, the main adverse reactions experienced by the patient were grade 2 hand and foot skin syndrome, grade 2 hypertension, and a slight reduction in FT3 and FT4 levels pertaining to thyroid function. The first two adverse events are common reactions to the targeted drug anlotinib. The alteration in thyroid function is considered to be immune-related hypothyroidism caused by toripalimab. After three cycles of the medication, the dosage of anlotinib was appropriately reduced to 10 mg/day. Simultaneously, synthetic thyroxine supplementation was given. Subsequently, the adverse reactions were alleviated, and the patient’s blood pressure returned to normal levels.

Nevertheless, intratumoral heterogeneity has remained a barrier to the efficacy of therapies. Intratumoral heterogeneity of the PD-1/PD-L1 expression also contributes to the diminishment of anticancer therapy in tumors (37). Grigg, C.M., et al. (38) reported that intratumoral heterogeneity of Human Epidermal Growth Factor Receptor 2 (HER2) in 44% of HER2-positive primary UC, which was associated with loss of HER2 in metastatic lesions. The intratumoral heterogeneity of ERBB2 amplification and HER2 expression has also been identified in micropapillary UC (39). Consequently, we infer that the intratumoral heterogeneity is also present in SUC, which may influence the ultimate efficacy of ICIs and TKIs. Fortunately, to date, the patient’s condition has been satisfactory.

### 3.4 Prognosis

Renal pelvis SUC is characterized by low cell differentiation, a high degree of malignancy, and rapid diffusion and metastasis. Post-surgically, abdominal implant metastasis is commonly encountered. The disease progresses rapidly, the prognosis is often worse than that of high-grade UC, and the survival period is <2 years (10, 17). Recurrence after surgery often results in death due to distant metastasis and organ failure. In this case, the patient developed intraperitoneal implantation and metastasis rapidly after the two surgeries, and the tumor spread rate was much higher than that of common UC. These clinical manifestations confirmed the characteristics of low differentiation and poor prognosis of SUC tumor cells. However, the other side of tumor cells with high malignancy and low differentiation is that if the treatment is effective, the effect is obvious. After the targeted immunotherapy took effect in our patient, the tumor cells were rapidly eliminated. As there is no standard treatment guide for reference, maintenance therapy is being continued to date. However, there is no standard for the duration of maintenance treatment. For most immune drugs in other cancers, maintenance therapy is recommended for one to two years. The use of targeted drugs is generally recommended until the disease progresses or adverse reactions become intolerable. The patient has undergone treatment for a period of two years, and the subsequent examination results indicated no signs of tumor recurrence. The patient’s overall condition is satisfactory with a commendable quality of life. Following the treatment protocol, medication has been discontinued and close follow-up will be done to adjust the treatment according to the patient’s condition.

Recently, some investigations recognized that variant histology (VH) in UC is a significant prognostic factor that can lead to more aggressive disease behavior and poorer survival rates. Specifically, VH is associated with an increased risk of metastasis after radical nephroureterectomy for upper tract UC and a higher likelihood of recurrence post-radical cystectomy for bladder cancer (40, 41). Certain VH subtypes, such as plasmacytoid and small-cell, are consistently linked to a worse disease-specific survival (42). SUC is likely to exhibit the characteristics of VH, representing a significant avenue for our forthcoming endeavors.

## 4 Conclusion

Renal pelvis SUC is a malignant tumor with high malignancy and strong invasion and tends to metastasize easily. Distinguishing the condition from common UC based on clinical manifestations is difficult. The diagnosis depends on histopathological examination and immunohistochemical analysis. In terms of treatment, radical surgery continues to be the most important treatment for patients with SUC who have surgery opportunities. The clinical benefits of radiotherapy and chemotherapy for patients with SUC are yet to be clarified. Targeted immunotherapy had a beneficial effect on our patient, which suggests that the use of ICIs combined with TKI may benefit patients with SUC. Although this report is based on a single case, immunotherapy or targeted therapy or a combination of both is a potential research direction.

## Data Availability

The original contributions presented in the study are included in the article/**Supplementary Material**. Further inquiries can be directed to the corresponding author.

## Supplementary material

Supplementary Figure 1 Renal MRI T2 images of the pelvic metastatic tumor before and after the combination therapy of toripalimab plus anlotinib (axial plane). **(A–I)** The examination time corresponds to that of each image with the same label in **Figure 3**. After six cycles of treatment, the pelvic metastatic tumor disappeared. MRI, magnetic resonance imaging.

Supplementary Figure 2 Renal MRI T2 images before and after the combination of toripalimab and anlotinib (coronal plane). **(A–I)** The examination time corresponds to that of each image with the same label in **Figure 3**. After six cycles of treatment, the tumor basically disappeared. MRI, magnetic resonance imaging.

Supplementary Figure 3 No signs of tumor recurrence were observed in the follow-up MRI results on May 8, 2024. MRI, magnetic resonance imaging.
