# Supplementary figures and images for: Case report: Toripalimab plus anlotinib in postoperative recurrent renal pelvic sarcomatoid urothelial carcinoma
Source: Front Oncol. 2024 Sep 25;14:1397855. doi: 10.3389/fonc.2024.1397855 (PMC11461166; doi:10.3389/fonc.2024.1397855)

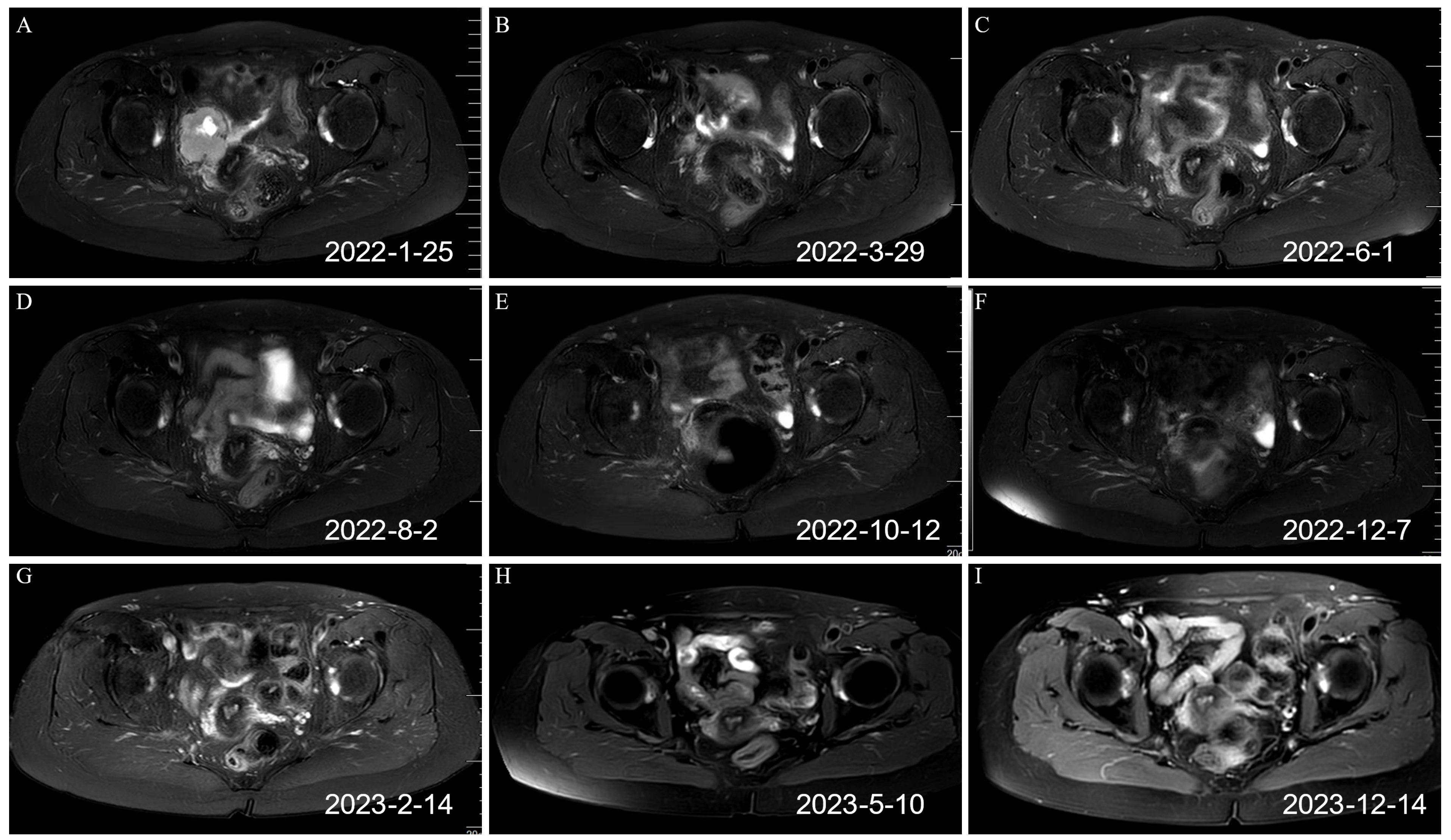

Supplement: Supplementary Figure 1 — Renal MRI T2 images of the pelvic metastatic tumor before and after the combination therapy of toripalimab plus anlotinib (axial plane). (A–I) The examination time corresponds to that of each image with the same label in Figure 3 . After six cycles of treatment, the pelvic metastatic tumor disappeared. MRI, magnetic resonance imaging. [file Image1.tif]

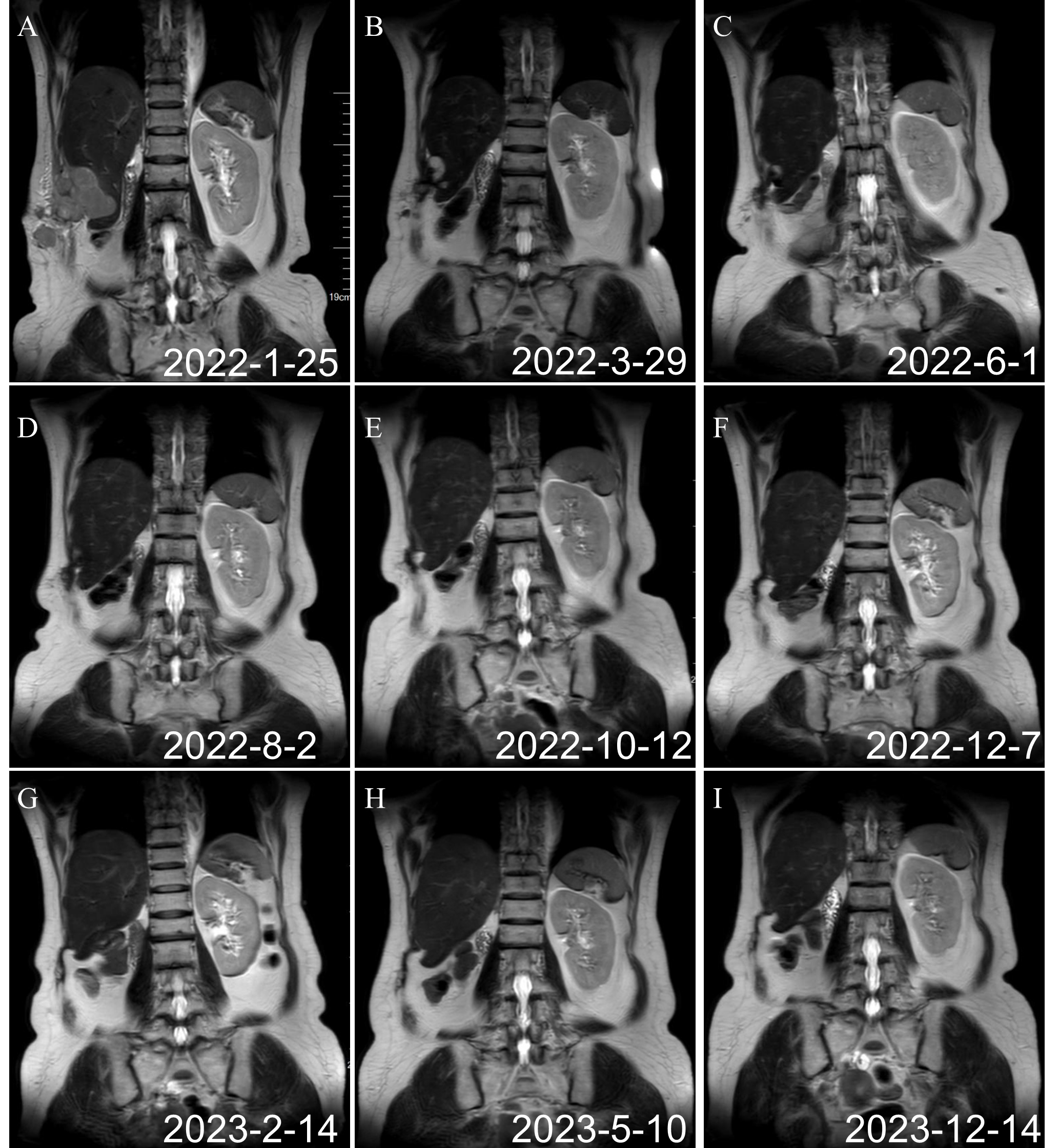

Supplement: Supplementary Figure 2 — Renal MRI T2 images before and after the combination of toripalimab and anlotinib (coronal plane). (A–I) The examination time corresponds to that of each image with the same label in Figure 3 . After six cycles of treatment, the tumor basically disappeared. MRI, magnetic resonance imaging. [file Image2.tif]

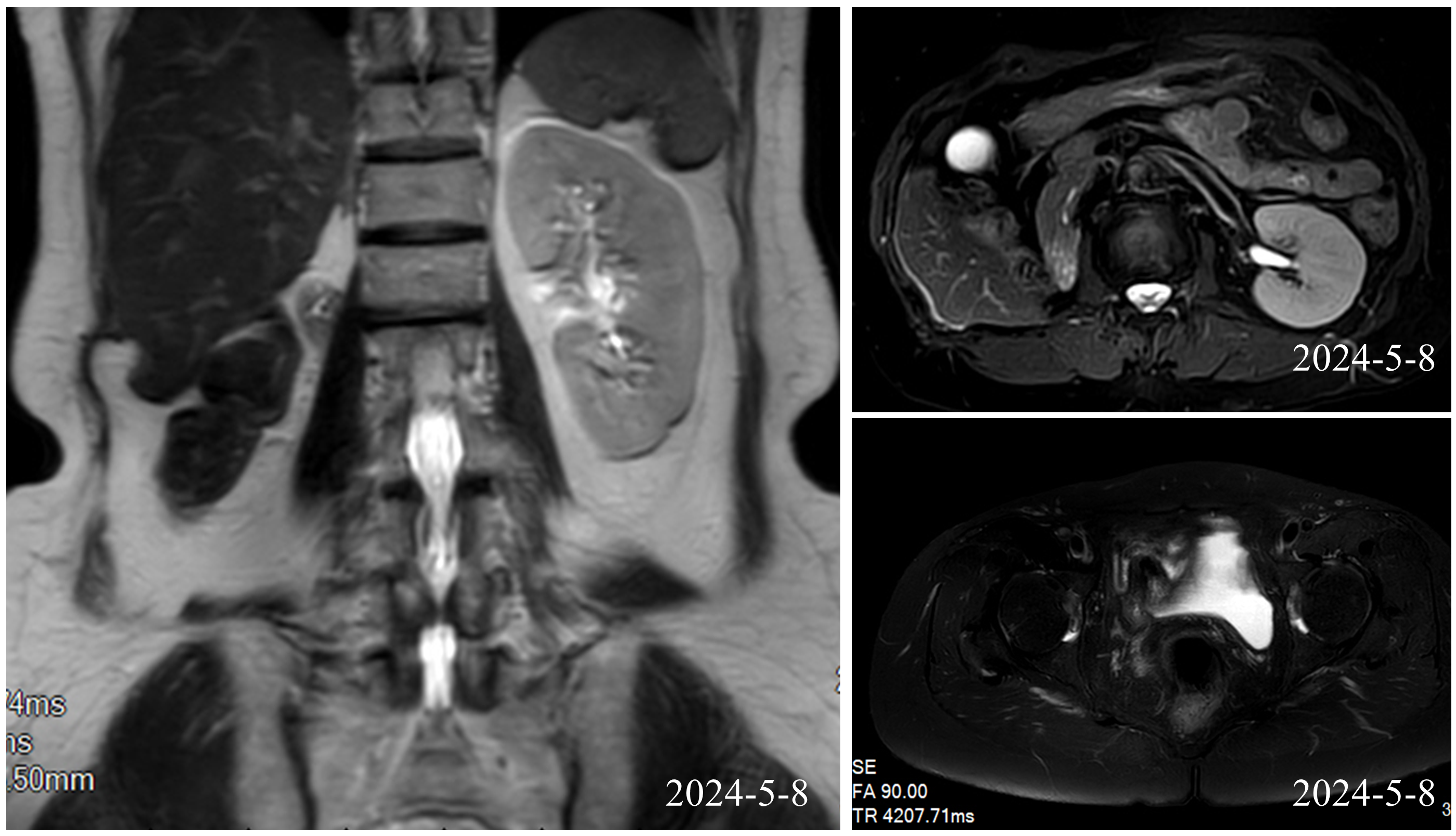

Supplement: Supplementary Figure 3 — No signs of tumor recurrence were observed in the follow-up MRI results on May 8, 2024. MRI, magnetic resonance imaging. [file Image3.tif]
